# Efficient HIV-1 *Trans* Infection of CD4^+^ T Cells Occurs in the Presence of Antiretroviral Therapy

**DOI:** 10.1093/ofid/ofz253

**Published:** 2019-05-24

**Authors:** Giovanna Rappocciolo, Nicolas Sluis-Cremer, Charles R Rinaldo

**Affiliations:** 1 Department of Infectious Diseases and Microbiology, Graduate School of Public Health, University of Pittsburgh, Pittsburgh, Pennsylvania; 2 Department of Medicine, University of Pittsburgh, Pittsburgh, Pennsylvania; 3 Department of Pathology, School of Medicine, University of Pittsburgh, Pittsburgh, Pennsylvania

**Keywords:** antigen-presenting cells, ART, B lymphocytes, dendritic cells, HIV, *trans* infection

## Abstract

**Background:**

Antiretroviral therapy (ART) has dramatically improved the quality of life of people with HIV-1 infection (PWH). However, it is not curative, and interruption of ART results in rapid viral rebound. Cell-to-cell transfer of HIV-1, or *trans* infection, is a highly efficient mechanism of virus infection of CD4^+^ T cells by professional antigen-presenting cells (APCs), that is, dendritic cells (DCs), macrophages, and B lymphocytes.

**Methods:**

APC from HIV seronegative donors treated with ART *in vitro* (CCR5 agonist, NRTI, PI and NNRTI, alone or in combination), were loaded with HIV R5-tropic HIV_Bal_ and mixed with autologous or heterologous CD4^+^ T lymphocytes to assess *trans* infection. Ex vivo APC from chronic HIV-infected MACS participants before and after initiation of ART, were also loaded with HIV R5-tropic HIV_Bal_ and tested for trans infection against autologous or heterologous CD4^+^ T lymphocytes. Virus replication was measured by p24 ELISA.

**Results:**

Here we show in vitro that antiretroviral drugs did not block the ability of DCs and B cells to *trans*-infect CD4^+^ T cells, although they were effective in blocking direct *cis* infection of CD4^+^ T cells. Moreover, ex vivo DCs and B cells from ART-suppressed PWH mediated efficient HIV-1 *trans* infection of CD4^+^ T cells, which were resistant to direct *cis* infection.

**Conclusions:**

Our study supports a role for HIV-1 *trans* infection in maintenance of the HIV-1 reservoir during ART.

The introduction of antiretroviral therapy (ART) more than 2 decades ago has dramatically improved the quality of life of people with HIV-1 (PWH), strikingly reducing HIV-1-related mortality and morbidity. Although ART restores peripheral blood CD4^+^ T-cell numbers and decreases HIV-1 viral load to undetectable levels, it is not curative, as interruption of ART typically results in rapid viral rebound [1]. This is due to the ability of HIV-1 to establish a replication-competent, latent viral reservoir in CD4^+^ T cells. Mechanisms that maintain this reservoir are incompletely understood [2]. Early events in mucosal transmission of HIV-1 can involve infection of myeloid dendritic cells (DCs) that capture virus and travel to draining lymph nodes, where they could transfer HIV-1 to CD4^+^ T–follicular helper cells and other CD4^+^ T-cell subsets known to harbor the virus [3]. Such cell-to-cell transfer of virus, termed *trans* infection, has been extensively described by us and others as a highly efficient mechanism of transfer of HIV-1 to CD4^+^ T cells by professional antigen-presenting cells (APCs), that is, myeloid DCs and macrophages [4–8] and B lymphocytes [9–11]. A similar but distinct form of HIV-1 *trans* infection occurs between CD4^+^ T lymphocytes [12–14], where the level of viral replication in the *trans*-infected T cells is lower than in T cells *trans*-infected by APCs [8, 11].

It has been speculated that HIV-1 *trans* infection occurs during ART [15], potentially acting as a stealth pathway for persistence of virus. However, few studies have addressed this hypothesis. A recent report showed that 2 antiretroviral drugs, tenofovir and raltegravir, were ineffective in blocking DC-mediated HIV-1 *trans* infection of CD4^+^ T cells in vitro [16]. Other studies have shown a reduced efficacy of early, less potent antiretroviral drugs on T-cell-to-T-cell *trans* infection with HIV-1 [12, 13, 17]. Here we investigated whether 2 types of APCs, that is, DCs and B lymphocytes, derived from PWH enrolled in the Multicenter AIDS Cohort Study (MACS) and under long-term, virus-suppressive ART, maintain the ability to *trans*-infect autologous CD4^+^ T cells. We first show that in vitro treatment of DCs and B cells derived from HIV-1-seronegative donors with antiretroviral drugs did not block their ability to *trans*-infect CD4^+^ T cells. Importantly, this was confirmed and extended by demonstrating that ex vivo DCs and B cells from ART-suppressed PWH were able to mediate highly efficient *trans* infection of CD4^+^ T cells that were relatively resistant to direct *cis* infection. Our study supports a role for HIV-1 *trans* infection in maintenance of the HIV-1 reservoir during ART.

## METHODS

### Ethics Statement

Biological samples were acquired and studied from consented individuals according to University of Pittsburgh International Review Board–approved protocols. All recruited participants were over the age of 18 and provided informed consent before sample collection or use.

### Participants

We studied 10 HIV-1 chronically infected participants of the Pittsburgh portion of the MACS who were receiving ART who had an undetectable viral load and CD4^+^ T-cell counts >500 cells/mm^3^ at the time of the study. Two HIV-1 nonprogressors (NPs) who chose to initiate ART were also studied. HIV-1-seronegative blood bank donors were used to test the effect of ART on *trans* infection in vitro. A standard HIV-1-seronegative donor was always tested in parallel with MACS participants as a control for assay performance.

### Cell Isolation and Culture

CD4^+^ T lymphocytes, B lymphocytes, and monocytes were positively enriched from freshly isolated or frozen peripheral blood mononuclear cells (PBMCs) from consented Pittsburgh MACS participants or anonymous blood bank donors using anti-CD4, CD19, or CD14 monoclonal antibody (mAb)–coated magnetic bead separation (Miltenyi Biotech), according to the manufacturer’s instructions. DCs were derived from monocytes by culture with 1000 U/mL of granulocyte-macrophage colony-stimulating factor (GM-CSF; Miltenyi Biotech) and 1000 U/mL of recombinant human interleukin 4 (rhIL-4;R&D Systems) for 5 days in AIM-V medium (Gibco). CD4^+^ T cells and B cells were activated for 48 hours with 10 U/mL of delectinated interleukin 2 (IL-2; Roche) and 2 ug/mL of phytohemagglutinin (PHA; Sigma) or 1000 U/mL of rhIL-4 (R&D Systems) and 0.1 ug/mL of CD40L (Enzo Life Sciences), respectively.

R5-tropic HIV-1^BaL^ purified from PM1 cells (obtained through the National Institutes of Health [NIH] AIDS Reagent Program, Division of AIDS, NIAID, NIH. Lusso et al [18]) was used for the *cis* and *trans* infection experiments. Virus stock titration and experimental HIV-1 Gag p24 measurements were acquired by ELISA using the HIV-1 p24 Antigen Capture Immunoassay kit (SAIC-Frederick), per the manufacturer’s instructions.

### ART Drugs

The following ART drugs were tested at the indicated final concentrations: maraviroc (1 μM), tenofovir (100 μM), rilpivirine (500 nM), and darunavir (500 nM).

### *Trans* and *Cis* Infection of CD4^+^ T Cells

#### Trans Infection

To measure *trans* infection, 1 × 10^6^ APCs were loaded with HIV-1^BaL^ at 10^–3^ multiplicity of infection (m.o.i.) in minimal volume for 2 hours at 37ºC, washed 3 times with cold medium, and co-cultured with autologous PHA/IL-2-activated CD4^+^ T cells at a 1:10 ratio in complete medium [11]. APCs were also exposed to ART drugs alone or in combination before use in the *trans* infection experiments.

#### Cis Infection

CD4^+^ T cells untreated or exposed to ART drugs were infected directly in *cis* with HIV-1^BaL^ at an m.o.i. of 10^–1^ to determine susceptibility to infection. Supernatants from the *trans* and *cis* cultures were sampled every 4 days and tested for HIV-1 Gag p24 by ELISA.

### Statistics

Data were analyzed by 1-way analysis of variance, followed by the Student t test. GraphPad prism 7.0 Software was used for statistical analysis.

## RESULTS

### APC-to-CD4^+^ T-Cell *Trans* Infection by HIV-1 Is Not Inhibited by ART In Vitro

**Figure 1.**
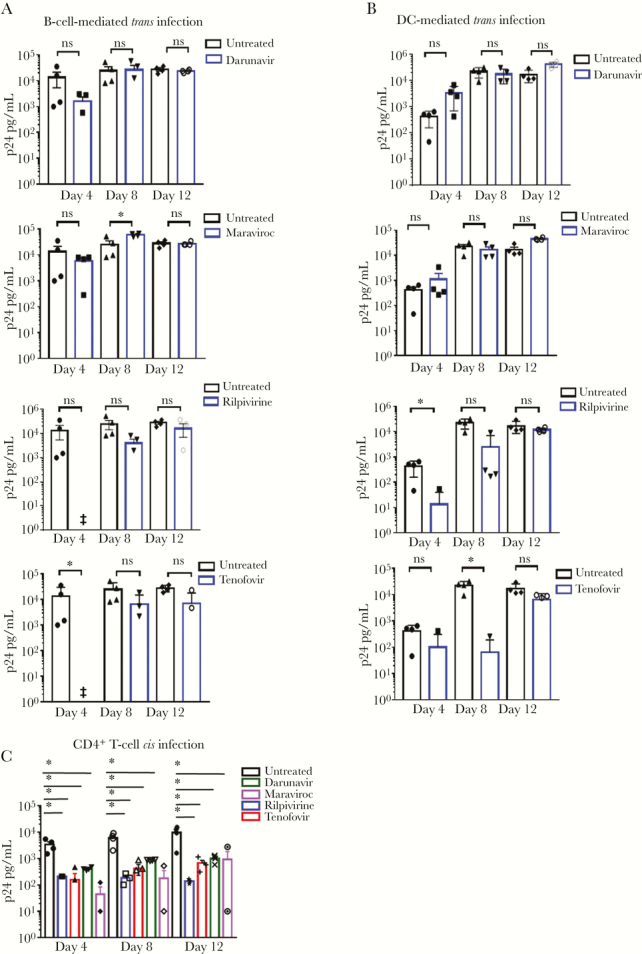
**Effect of antiretroviral drugs on B cell and DC mediated *trans* infection *in vitro*. Panel A and B.** B cells or DC were treated with the indicated drugs, loaded with HIV^Bal^ at 10^-3^ m.o.i. and mixed with activated, purified autologous CD4^+^ T cells as described in Methods. **Panel C.** Cell-free *cis* infection of CD^+^ T cells with HIV^Bal^ at 10^-1^ m.o.i. was conducted in parallel to determine CD^+^ T cells susceptibility to infection. Cell culture supernatants were collected at the indicated time points and HIV-1 Gag p24 was measured by ELISA. Data are mean values ±SE; n=4 experiments. GraphPad prism 7.0 Software was used for statistical analysis (one-way ANOVA followed by Students t test. *p<0.05) ‡= below limit of detection. Abbreviation: ns, nonsignificant.

We previously reported [10, 11] that HIV-1 *trans* infection of CD4^+^ T cells by DC and B cells results in highly efficient virus replication in CD4^+^ T cells, as measured by levels of HIV-1 Gag p24 over 12 days of co-culture. Here we set out to determine the effect of ART drugs on APC-mediated cell-to-cell HIV-1 *trans* infection. We first exposed B cells, DCs, and CD4^+^ T cells obtained from healthy, de-identified, HIV-1-negative blood donors to the protease inhibitor (PI) darunavir, the nucleoside reverse transcriptase inhibitor (NRTI) tenofovir, the non-nucleoside reverse transcriptase inhibitor (NNRTI) rilpivirine, or the CCR5 co-receptor-blocking agent maraviroc (Figure 1A, B, and C, respectively). All drugs were used at concentrations that blocked direct HIV-1 *cis* infection of CD4^+^ T cells (Figure 1C). We used HIV-1 Gag p24 levels in culture supernatants over time as a measure of productive infection in CD4^+^ T cells. Therefore, only levels of infectious, replicating HIV-1 were measured. CD4^+^ T cells from the same donors were also tested for their susceptibility to HIV-1 *cis* infection, either in the presence of ART drugs or when left untreated. Finally, the amount of input virus loaded into DCs and B cells for assessing *trans* infection was chosen to be highly inefficient for productive infection of CD4^*+*^ T cells in *cis* [8, 10, 11]. This allowed us to focus on the levels of p24 measured in the APC-T-cell co-cultures resulting from *trans* infection of CD4^+^ T cells.

As shown in Figure 1 A, B-cell-mediated *trans* infection of CD4^+^ T cells was not significantly affected over 12 days by exposure to the drugs tested. Among the drugs tested, treatment with darunavir and maraviroc did not affect the ability of both B cells and DCs to efficiently transfer infectious, replicating virus to the target CD4^+^ T cells. Only rilpivirine and tenofovir treatment significantly reduced the efficiency of *trans* infection at the earlier time points, although we were still able to detect HIV-1 Gag p24 production by day 12 in culture comparable to that in untreated cultures.

Notably, treatment with maraviroc, a CCR5-blocking agent, significantly enhanced *trans* infection of target CD4^+^ T cells by 8 days of co-culture when B lymphocytes were used, but this effect was not detected at day 12 (Figure 1A). Maraviroc has been reported to increase CCR5 expression on CD4^+^ T cells in both humans and macaques [19, 20], suggesting that it could actually increase the susceptibility of T cells to HIV-1 infection. Interestingly, we did not observe a maraviroc-enhancing effect on *trans* infection in the DC-CD4^+^ T-cell co-cultures, which was present when B cells were used as APCs.

**Figure 2.**
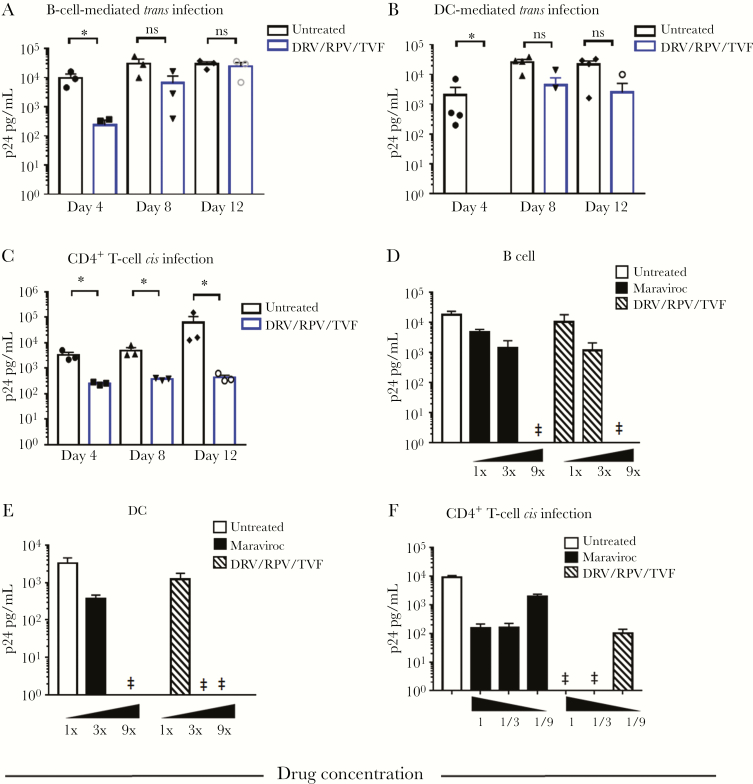
**Effect of combination antiretroviral drugs on B cells and DC mediated *trans* infection in vitro.** B cells (**panel A**) or DC (**panel B**) were treated with the indicated drugs in combination, loaded with HIV^Bal^ at 10^-3^ m.o.i. and mixed with activated, purified autologous CD4^+^ T cells. Purified CD4^+^ T cells were also infected in *cis* in the presence of the drug combination (**panel C**). Drug concentrations are described in Methods. Data are mean values ±SE; n=4 experiments. **Panel D and E**: B cells and DC were treated with up to 9 times the concentration of maraviroc or of the three drug combination, respectively and used in *trans* infection experiments as described above. **Panel F**: purified CD4^+^ T cells were also infected with cell free virus in the presence of maraviroc or of the three drug combination in decreasing concentrations. Cell cultures supernatants were collected at the indicated time points and HIV-1 Gag p24 was measured by ELISA. Data are representative of 2 independent experiments and are mean values ±SE of triplicate wells. GraphPad prism 7.0 Software was used for statistical analysis (one-way ANOVA followed by Students t test. *p<0.05) ‡= below limit of detection. Abbreviations: DC, dendritic cell; DRV/RPV/TVF, darunavir/rilpivirine/tenofovir.

Considering that most ART regimens are now comprised of >1 drug, we tested the efficiency of *trans* infection in the presence of a 3-drug combination, that is, darunavir/rilpivirine/tenofovir (DRV/RPV/TVF). As shown in Figure 2, efficient *trans* infection occurred even in the presence of the DRV/RPV/TVF combination using either DCs or B cells as APCs (Figure 2A and B, respectively). As a comparison, the effect of the TVF/RPV/DRV combination on effectively reducing cell-free *cis* infection of CD4^+^ T cells is shown in Figure 2C.

It has been postulated that the lack of effect of ART on *trans* infection is a consequence of high virus particle delivery at the cell-to-cell juncture [13, 21]. Therefore, we tested whether HIV-1 *trans* infection could be blocked by increasing the drug concentrations to overcome the *trans* infection effect. As shown in Figure 2D and E, increasing drug concentrations up to 9 times the level needed to block *cis* infection resulted in suppression of HIV-1 *trans* infection by B cells and DCs, respectively. By the same token, reducing the drug concentration up to 9 times allowed for *cis* infection of CD4^+^ T cells (Figure 2F). Taken together, these data show that APC-mediated, efficient *trans* infection of T cells with HIV-1 can take place in the presence of ART drugs and can be overcome only by high ART concentrations.

### Ex Vivo APCs From Participants on ART Can *Trans*-Infect CD4^+^ T Cells

**Figure 3.**
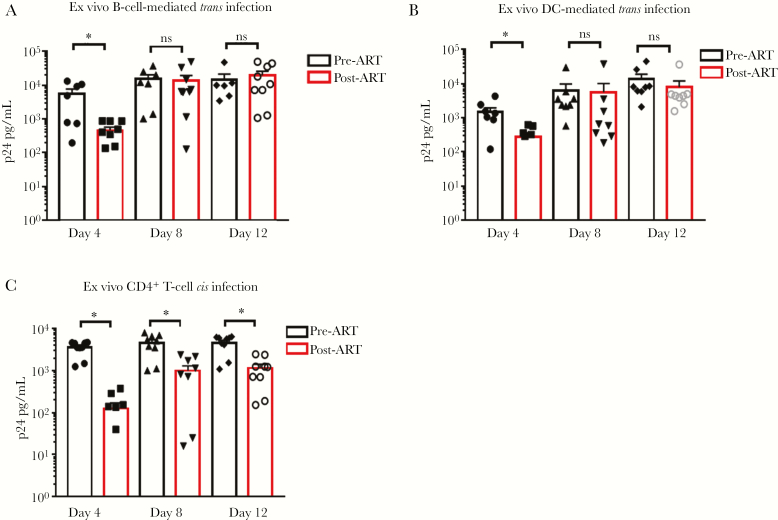
**Ex-vivo APC from ART suppressed patients *trans* infect CD4^+^ T cells**. B cells (**panel A**) and DC (**panel B**) derived from participants under suppressive ART (black bars) were loaded with HIV^Bal^ at 10^-3^ m.o.i as described in Methods and co-cultured with autologous CD4^+^ T cells for 12 days. B cells and DC derived from archived PBMC from participants prior to ART initiation were also tested in parallel (red bars). Supernatants were collected at the indicated time points and tested for HIV-1 Gag p24. Data are mean values ±SE; n=10 experiments. **Panel C.** Purified CD4^+^ T cells from the participants described in panels A and B were infected *in cis* with HIV^Bal^ at 10^-1^ m.o.i. in parallel with CD4^+^ T cells archived prior to ART initiation. Supernatants were collected at the indicated time points and tested for HIV-1 Gag p24. Data are mean values ±SE; n=10 experiments. GraphPad prism 7.0 Software was used for statistical analysis. *p<0.05. Abbreviations: ART, antiretroviral therapy; DC, dendritic cell.

We next addressed whether APCs derived from HIV-1-infected individuals on virus-suppressive ART can *trans*-infect autologous CD4^+^ T cells ex vivo. We tested 10 participants under suppressive ART and found that B cells and DCs derived from these individuals mediated efficient *trans* infection of autologous CD4^+^ T cells compared with *trans* infection mediated by APCs archived before therapy initiation (Figure 3A and B, respectively). We also determined if potential ex vivo carryover of antiviral drug activity could affect direct *cis* infection of CD4^+^ T cells, comparing PBMCs obtained from the same MACS participants before and after initiation of ART. As shown in Figure 3C, CD4^+^ T cells post-ART were not able to support HIV-1 replication as well as T cells from pre-ART blood, indicating that the in vivo antiviral effect of their ART carried over ex vivo. Even in the face of this antiviral effect on the CD4^+^ T cells ex vivo, we found that both DCs and B cells of HIV-1-infected participants on ART were able to *trans-*infect autologous CD4^+^ T cells derived from the same blood samples (Figure 2A and B, respectively) with levels of HIV-1 Gag p24 at least 1 log_10_ higher than by *cis* infection (Figure 2C). As we observed in seronegative participants, B-lymphocyte-mediated *trans* infection resulted in higher levels of virus replication compared with DCs. Thus, APCs from participants on virus-suppressive ART and tested ex vivo can efficiently mediate HIV-1 *trans* infection of CD4^+^ T cells that are resistant to direct *cis* infection.

**Figure 4.**
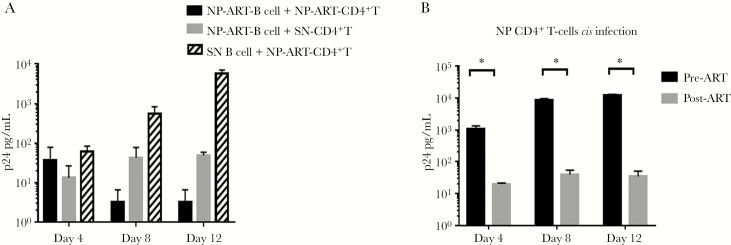
**APC from NP do not *trans* infect CD4^+^ T cells**. B cells from NP under ART were loaded with HIV^Bal^ at 10^-3^ m.o.i. and mixed with autologous (black bar) or heterologous, CD4^+^ T cells from SN donor (grey bar). CD4^+^ T cells from NP under ART were co-cultured with B cells from a SN donor loaded with HIV^Bal^ at 10^-3^ m.o.i ( thatched bar) (B) Purified CD4^+^ T cells from NP were infected in cis with HIV^Bal^ at 10^-1^ m.o.i. in parallel with CD4^+^ T cells cryopreserved prior to ART initiation. Supernatants were collected at the indicated time points and tested for HIV-1 Gag p24. Data are representative of 2 independent experiments and are mean values ±SE of triplicate wells. GraphPad prism 7.0 Software was used for statistical analysis. *p<0.05. Abbreviations: ART, antiretroviral therapy; NP, nonprogressor; SN, HIV-1-seronegative donor.

We have previously shown that APCs from PWH who are able to control disease progression in the absence of ART, that is, NPs, are unable to *trans*-infect autologous and heterologous CD4^+^ T cells and that this phenotype is under the control of cellular cholesterol homeostasis regulation [10]. Here we tested 2 NPs who chose to initiate ART to determine if this phenotype was maintained during therapy. As shown in Figure 4, B cells derived from NPs before or after initiation of ART (NP-ART) were unable to *trans*-infect autologous CD4^+^ T cells or heterologous CD4^+^ T cells obtained from an HIV-1-seronegative donor (SN). B cells from the SN donor were able to *trans*-infect CD4^+^ T cells from the NP-ART participant (Figure 4A). As expected, NP CD4^+^ T cells were able to support *cis* HIV-1 infection (Figure 4B) before ART but not after ART initiation. In contrast, APCs from HIV-1 progressors (PRs) on effective ART were able to *trans*-infect CD4^+^ T cells both before and after ART initiation, as shown in Figure 3A and B. CD4^+^ T cells from PRs were able to support *cis* HIV-1 infection before ART, but to a significantly lesser degree after ART initiation (Figure 3C). Taken together, these data demonstrate that the inability of APCs from NPs and the ability of APCs from PRs to *trans*-infect T cells ex vivo are maintained under highly effective ART.

## DISCUSSION

Here we show that HIV-1 *trans* infection of CD4^+^ T cells by 2 types of APCs, DCs and B lymphocytes, is insensitive to virus-suppressive levels of ART, that is, darunavir, rilpivirine, and maraviroc; rilpivirine, and to some extent tenofovir treatment in vitro, showed a limited effect. This was demonstrated both by in vitro treatment of these APCs from HIV-1 SN donors with concentrations of ART drugs sufficient to block *cis* infection and by inefficient HIV-1 *trans* infection mediated by APCs derived from the peripheral blood of HIV-1-infected individuals on suppressive ART. The concept that ART has a limited effect on *trans* infection by HIV-1 has been a long-standing concern regarding the efficacy of ART, primarily in terms of T-cell-to-T-cell infection [12, 13, 22, 23]. To our knowledge, there have not been comprehensive studies on the effects of ART on APC-to-T-cell *trans* infection [15, 24, 25], although a recent report showed that 2 formulations of current ART drugs, that is, tenofovir and raltegravir, administered in vitro failed to inhibit DC-to-T-cell *trans* infection [16]. Thus, our analysis is the first both to assess the effects of multiple types of ART on APC *trans* infection in vitro and ex vivo and to emphasize the importance of this being a stealth mode to circumvent the antiviral effects of ART.

Our study supports that APC-mediated *trans* infection of T cells with HIV-1 yields a high level of virus replication. The burst of virus production resulting from *trans* infection of target cells can be up to 1000-fold higher than that resulting from HIV-1 *cis* infection through passive dissemination in the extracellular fluid [26]. This has also been observed in time-lapse videos documenting the transmission of multiple viral particles at the point of contact between cells [27]. In fact, we regularly observed a 20- to 100-fold-greater level of HIV-1 replication mediated by DCs and B cells compared with direct *cis* infection of either autologous or heterologous CD4^+^ T cells, using a relatively low input multiplicity of HIV-1 (m.o.i. 10^–3^) compared with the 100-fold higher amount of HIV-1 used to demonstrate *cis* infection (m.o.i. 10^–1^). Interestingly, using colonic explant models showed that cell-associated HIV-1 in the mucosa is transmitted at a much higher efficiency compared with cell-free virus [28] and that myeloid DCs in human cervical explants are the first cells to capture HIV-1 and transfer it to mucosal CD4^+^ T cells [3].

The high efficiency of cell-to-cell HIV-1 infection is thought to be due to the formation of the virologic synapse, a virus-orchestrated contact between a cell carrying infectious virus and an uninfected target cell, which can overcome the target cell barriers that are active against cell-free HIV-1 infection [14, 29, 30]. Others have shown a lack of ART effect when *trans* infection is mediated by an APC [16, 21, 31], although these were in vitro studies. It is not clear if this phenomenon relates to HIV-1 replication in the APCs or to high virus particle transfer from these APCs to the T cells. A role for virus replication in *trans* infection by APCs is not likely the case for B lymphocytes, as they do not support productive HIV-1 replication [11]. However, CD40L-activated B cells can take up HIV-1 via the intercellular adhesion molecule-3–grabbing nonintegrin (DC-SIGN), which is essential for their *trans* infection of T cells [11]. Our data show that HIV-1 replication is not necessary in APCs for productive *trans* infection of CD4^+^ T cells, given the more efficient B-cell-mediated *trans* infection compared with DC-mediated *trans* infection, confirming previous observations [8, 11]. In contrast, HIV-1 can infect DCs through DC-SIGN or CD4 and chemokine receptors and lead to a limited, low level of virus production or no evidence of virus replication [24].

Although the efficiency of cell-to-cell transmission of HIV-1 is well established, its importance in the pathogenesis of HIV-1 is uncertain. There is conflicting evidence that viral persistence is achieved in persons on ART by ongoing virus replication in lymphoid tissues, with full HIV-1 suppression in the peripheral blood [13, 32–36]. Our current and previous results [8, 10, 11] argue for a pivotal role of APC-mediated *trans* infection in the control of HIV-1 disease progression and their role in the maintenance of the viral reservoir. The recognized importance of HIV-1 sequestration in B-cell follicles further supports a role for these APCs in maintaining virus infection through *trans* infection during the normal process of antigen presentation, thereby transferring infectious virus with high efficiency [7, 37].

Data presented here strengthen the notion that *trans* infection can be a potent mechanism of HIV-1 persistence in the B-cell follicles of lymphoid tissues [38], where target cells are in close proximity, allowing for the formation of APC-target cell contact [39]. Here we show for the first time that B-lymphocyte transfer of HIV-1 to CD4^+^ T cells is not susceptible to ART and that B cells derived from PWH under fully suppressive ART still maintain the ability to *trans*-infect autologous CD4^+^ T cells. Thus, myeloid DCs could mediate HIV-1 *trans* infection during their normal interaction with and signaling of immune responses in CD4^+^ follicular helper T cells (Tfh). Likewise, B lymphocytes normally signal Tfh to initiate antibody responses in B-cell follicles [40]. When these APCs are infected with HIV-1, this could create a constant supply of infected, virus-replicating target T cells in a privileged lymphoid compartment. There, ART drugs have limited access [32] and thus may not reach the necessary concentrations to overcome the high virus particle concentration at the site of cell-to-cell transfer [13, 14]. In tissues where target cells and APCs are in close contact, sufficiently high concentrations of HIV-1 are likely to be achieved, allowing for efficient spread and ART evasion.

Notably, APCs from HIV-1 NPs who have chosen to undergo ART maintained their inability to transfer virus to CD4^+^ T cells, with or without ART. In this regard, our recent data on the role of APCs’ cholesterol homeostasis in HIV-1 disease progression could inform effective strategies as an adjuvant to ART. Interestingly, a recent study showed that concomitant use of statins after treatment interruption is associated with lower risk of virologic rebound [9]. Taken together, these data highlight the pivotal importance of APC-mediated cell-to-cell spread of HIV-1 in the face of effective ART measured as viral suppression in the periphery. This stresses the need for a more comprehensive approach to the eradication of reservoirs, where interference with APC function could provide an important tool in thwarting efficient HIV-1 spread.
